# Structure-Enhanced Underwater Object Detection via Wavelet-Edge Collaboration and Selective Multi-Scale Fusion

**DOI:** 10.3390/s26103234

**Published:** 2026-05-20

**Authors:** Dejun Li, Chunrong He, Peng Tu, Shenshen Yang, Xinbei Lv, Jianjun Liu

**Affiliations:** 1 State Key Laboratory of Deepsea Manned Vehicle, China Ship Scientific Research Center, Wuxi 214082, China; lidj702@163.com (D.L.); hechunrong@cssrc.com.cn (C.H.); yss9158@aliyun.com (S.Y.); 2 School of Artificial Intelligence and Computer Science, Jiangnan University, Wuxi 214122, China; 18770155834@163.com; 3 College of Mechanical and Electrical Engineering, Harbin Engineering University, Harbin 150001, China; lvxinbei@hrbeu.edu.cn

**Keywords:** underwater object detection, WEC-UOD, wavelet subband compensation, edge-guided spatial correction, scale-selective fusion

## Abstract

Underwater object detection is important for ocean exploration and marine applications. However, underwater images are often degraded by absorption, scattering, and background interference, which weaken object contours, blur boundaries, and obscure fine texture details, thereby increasing the difficulty of detecting small objects and objects with large shape variations. To address these challenges, we propose WEC-UOD, an underwater object detector that improves structure-sensitive representation learning and multi-scale feature fusion within the detector, without relying on a separate image enhancement stage. In the backbone, the Wavelet–Edge Collaboration (WEC) module first uses wavelet-subband guidance to compensate for degraded structural and texture information and then applies edge-guided spatial correction to refine object boundaries and local geometry. In the neck, the Scale-Selective Fusion (SSF) module adaptively selects informative responses from branches with different receptive fields and further suppresses background interference through channel and spatial recalibration. Experiments on RUOD and DUO show that WEC-UOD achieves mAP@0.5 scores of 87.4% and 86.9%, respectively, consistently outperforming the YOLOv11s baseline. These results demonstrate the effectiveness of combining structural enhancement with selective multi-scale aggregation for underwater object detection.

## 1. Introduction

Underwater object detection (UOD) is important for marine ecological monitoring, resource exploration, and autonomous underwater operations. However, compared with terrestrial scenes, underwater optical images are more easily degraded by light scattering, selective absorption, and non-uniform reflection, which often lead to low visibility, low contrast, blurred boundaries, color distortion, and background noise. These factors substantially increase the difficulty of reliable object detection in underwater environments [1,2].

To address these challenges, many existing studies attempt to improve input quality by introducing underwater image enhancement (UIE) as a preprocessing step. Nevertheless, the optimization objectives of image enhancement and object detection are not fully aligned. In practice, enhanced images do not always translate into consistent gains in detection accuracy and may even impair the robustness of high-level vision tasks by introducing artifacts or causing distribution shift [3]. Therefore, rather than relying on an independent enhancement module, directly improving feature representation and adaptability within the detection network itself under degraded underwater conditions has emerged as a more promising research direction [4].

From the perspective of internal representation learning in detectors, a central challenge caused by underwater degradation lies in the continuous attenuation of object structural information during feature extraction. For small underwater objects and objects with large shape variations, fine-grained details such as contours, boundaries, and weak textures are essential for accurate localization and category discrimination. However, under complex conditions characterized by low contrast, blurred edges, and coupled background interference, such structure-sensitive information is often difficult to preserve effectively.

Meanwhile, underwater scenes often contain heavy background clutter caused by suspended particles, ripple reflections, and uneven illumination. These factors can easily induce target-irrelevant false responses and thereby interfere with the feature learning process [5]. If the detector relies only on conventional convolutional stacking and downsampling operations, the local structural details of small objects are highly susceptible to further loss during semantic abstraction [6]. At the same time, background noise may accumulate and be progressively amplified across layers, ultimately leading to localization errors and missed detections.

In view of this, some recent studies have attempted to enhance structural representation under degraded conditions by introducing edge cues and frequency-domain decomposition mechanisms [2]. Edge information helps preserve object contours and enhance local geometric patterns, while frequency-domain decomposition provides a distinctive perspective for disentangling appearance degradation from structural degradation [7,8]. In particular, wavelet transform offers a solid representational basis for recovering and reconstructing structural content in degraded scenes, owing to its ability to separate low-frequency contour components and high-frequency detail components across multiple scales [9,10]. Therefore, how to effectively combine frequency-domain decomposition with edge priors to recover robust structural representations in complex underwater scenes remains an important problem in underwater object detection.

Beyond the weakening of structural cues, the limitations of multi-scale feature fusion also constitute a major bottleneck in underwater small object detection. Small object detection relies heavily on shallow spatial details, whereas robust semantic recognition depends on high-level semantics. For this reason, feature pyramid networks (FPNs) and their variants have been widely adopted for multi-scale feature integration [11]. However, in complex underwater scenes, conventional fusion strategies based on fixed paths or non-selective aggregation often propagate informative target features and background noise indiscriminately. Such undifferentiated propagation not only leads to insufficient semantic alignment across features of different scales, but also aggravates the dilution of small-object information during the fusion process [6,11]. Therefore, multi-scale fusion in degraded underwater scenes should move beyond simple cross-level interaction. A more suitable strategy is to first select informative responses across scales and then recalibrate the fused representation, so that small-object features can be preserved while the propagation of background noise is suppressed.

Related studies on complex imaging tasks have also explored prior-guided modeling and multiscale representation learning under ill-posed or degraded conditions [12,13], suggesting that enhancement of structure-sensitive representations and feature aggregation can be beneficial beyond a single imaging domain. Motivated by the above observations, this study addresses two issues in underwater object detection: the degradation of structure-sensitive representations during feature extraction and the insufficient selectivity of multi-scale feature fusion in complex underwater scenes. To this end, we build WEC-UOD on YOLOv11 [14]. The proposed framework improves detector-internal representation learning under underwater degradation without relying on an independent image enhancement stage. Specifically, WEC is introduced into the backbone to recover structure-sensitive features through frequency-domain compensation followed by edge-guided spatial refinement, while SSF is incorporated into the neck to select informative multi-scale responses before channel–spatial recalibration. The main contributions of this study are summarized as follows:

(1) We design a Wavelet–Edge Collaboration (WEC) module with a sequential compensation-correction mechanism for backbone feature enhancement. WEC first uses wavelet-subband guidance to compensate degraded structural and texture responses, and then applies edge-guided spatial refinement on the compensated representation.
(2) We propose a Scale-Selective Fusion (SSF) module for neck feature aggregation. SSF adaptively selects responses from branches with different receptive fields and refines the fused representation through lightweight channel–spatial recalibration.
(3) By integrating WEC and SSF into YOLOv11, we build WEC-UOD and evaluate it on the RUOD and DUO datasets. Experimental results show that the proposed method consistently improves the YOLOv11s baseline and achieves competitive performance in underwater object detection.

## 2. Related Work

### 2.1. Underwater Object Detection and YOLO-Based Methods

Recent UOD research has gradually shifted from preprocessing-based enhancement to detector-oriented modeling, largely because independent UIE modules often bring limited and inconsistent gains for downstream detection. Despite this shift, most existing methods are still developed on top of general-purpose object detection frameworks [15]. Among them, YOLO-based one-stage detectors have become a widely adopted paradigm in UOD due to their advantages in inference speed and deployment efficiency [16]. However, directly transferring such detectors to underwater scenes remains highly challenging. Low contrast caused by light scattering, the dense distribution of small objects, and substantial scale variation make it difficult for conventional backbones to learn sufficiently discriminative features, often resulting in degraded detection performance [17,18].

To address these issues, researchers have made targeted improvements to backbone design, multi-scale fusion in the neck, and detection heads. For example, TC-YOLO incorporates Transformer self-attention and Coordinate Attention into the YOLO framework to enhance the discriminative representation of small objects while maintaining a balance between model complexity and deployment requirements [19]. Chen et al. proposed Dynamic YOLO [8], which reconstructs the backbone with deformable convolution to improve adaptive feature extraction for irregular object deformations. They further designed a dynamic feature fusion network and an improved decoupled detection head, thereby alleviating insufficient alignment across multi-scale representations as well as the conflict between classification and localization tasks. Zhang et al. [9] introduced a dedicated small-object prediction head based on YOLOv8 and combined deformable convolution with an attention mechanism during feature fusion, significantly improving the detection accuracy of dense and irregularly shaped small objects.

Although these YOLO-based improvements have enhanced underwater detection performance to some extent through architectural refinement, their feature representations and multi-scale fusion quality remain limited under challenging conditions such as strong background interference, weak-texture targets, and the rapid attenuation of small-object features. In particular, existing studies still lack a unified design that jointly preserves degradation-sensitive structural cues and selectively integrates multi-scale features for small-object detection in underwater scenes.

### 2.2. Frequency-Domain and Edge-Based Methods for Degraded Underwater Vision and Object Detection

In addition to detector-oriented methods, low-level enhancement and dehazing methods for degraded images have also provided important insights into underwater vision. Early studies mainly relied on fusion strategies and prior-guided restoration to improve visibility, contrast, and color fidelity in degraded scenes [20,21]. More recent restoration studies for degraded images have further explored physically motivated dehazing priors, as exemplified by IHDCP, which models transmission through an inverted haze density correction prior [22]. Benchmark-driven learning-based methods then promoted underwater image enhancement by introducing large-scale real-world datasets and dedicated restoration networks [23]. In parallel, physically guided and frequency-aware deep models, such as Ucolor and the wavelet-based physically guided normalization network for real-time dehazing, have shown that combining transmission-related modeling with feature-domain frequency decomposition is effective for recovering degraded structures and low-contrast details [24,25]. Although these methods are mainly designed for image-level restoration rather than object detection, they still highlight the importance of recovering degraded appearance and structural cues for robust underwater vision.

Underwater imaging degradation is not limited to statistical distortions in color and illumination; more fundamentally, it manifests as blurred boundaries, weakened textures, and the loss of object structural information. To address such mixed degradations, researchers have gradually recognized that spatial-domain modeling alone is insufficient to balance appearance restoration and structure preservation. As a result, frequency-domain decomposition, structural reconstruction, and boundary-preserving mechanisms have emerged as important research directions.

These methods were initially developed mainly for underwater image enhancement, with the aim of improving visual quality and recovering structural details in degraded images. For example, Jamadandi [26] incorporated wavelet transform into a network and exploited its ability to achieve accurate signal reconstruction in order to handle the complex nonlinear distortions caused by selective absorption and scattering. Huo et al. [27] further pointed out from the perspective of spectral distribution that color and illumination distortions in underwater images are mainly concentrated in low-frequency components, whereas the degradation of edges and textures is primarily reflected in high-frequency components. Based on this observation, they adopted a wavelet decomposition strategy to enhance high- and low-frequency subbands differently, enabling targeted treatment of mixed degradations. Subsequently, methods such as U-ENHANCE [28] deeply integrated wavelet-based frequency decomposition with attention mechanisms, highlighting the complementarity between frequency-domain analysis and spatial modeling and effectively preserving geometric integrity while restoring structural details.

Inspired by recent advances, frequency-domain modeling has gradually been introduced for degradation-aware feature optimization within the detector [29]. From a detection-oriented perspective, F3M [30] proposed a frequency-domain feature fusion module that explicitly distinguishes low-frequency appearance degradation from high-frequency structural cues, further indicating that frequency-domain modeling has extended from image enhancement to internal representation optimization in detectors. The creators of SFUDNet [31] observed the differences in frequency-domain distributions across underwater datasets and developed a spatial–frequency collaborative modulation framework, which significantly improved robustness against blur, noise, and color distortion. UEAOD [32] combined a joint enhancement-detection framework with frequency-domain decomposition, enabling the explicit separation of low-frequency appearance components and high-frequency structural components within the detection network, and further employed a dual-stream context fusion network for representation reorganization. The creators of UDINO [33] further linked frequency-domain modeling with degradation suppression, arguing that water scattering mainly weakens high-frequency textures and edge information. Based on this observation, they introduced a multi-scale high-frequency enhancement strategy to alleviate blur and adopted a low-frequency gating mechanism to suppress interference from scattering and color distortion in feature channels. In addition, FIOD-VUE [34] demonstrated, from the perspective of cross-environment generalization, the importance of frequency-domain modeling for extracting stable and invariant features.

In addition to frequency-domain information, edge and structural priors also provide valuable guidance for improving underwater detection performance. To address the difficulty of separating targets from the background under blurred underwater boundaries, some studies have begun to explicitly introduce edge cues into detection networks. For example, ERL-Net [35] enhances discriminative object representations through edge-guided attention and feature aggregation mechanisms. GEE-UOD [5], by contrast, improves feature extraction in complex environments through the collaborative enhancement of global context and edge information.

Existing studies indicate that frequency-domain modeling is effective for recovering degradation-sensitive structural details, while edge priors are useful for sharpening target boundaries and suppressing local interference. However, these two strategies are usually introduced separately. Effectively coupling frequency compensation with edge-guided refinement within a unified detector module remains underexplored.

### 2.3. Multi-Scale Feature Fusion and Scale-Adaptive Strategies

Small object detection has long been a fundamental challenge in computer vision. In underwater scenes, this problem is further exacerbated by the combined effects of blur, low contrast, and scattering noise: fine-grained details in shallow features are highly susceptible to being lost during downsampling and semantic abstraction [36], while deep features often struggle to form stable semantic representations because the target responses are severely weakened. Although FPNs and their variants [37] have been widely adopted for multi-scale feature integration, simple feature aggregation in underwater scenes often introduces a large amount of background noise from shallow details, whereas high-level semantics are still unable to effectively guide the screening of shallow features. Therefore, multi-scale fusion in underwater detection should not be treated as a mere stacking of features. More importantly, it should achieve a proper balance among detail compensation, semantic alignment, and noise suppression during cross-level interaction. Related studies on high-dimensional image analysis have also shown that model-driven representation design can improve feature decoupling and reconstruction quality in complex imaging tasks [38]. Although these studies are not focused on underwater object detection, they provide additional methodological support for adaptive feature organization under degraded imaging conditions.

To achieve this goal, existing studies have mainly explored two directions: cross-scale feature enhancement and fusion-topology optimization. Some works strengthen information interaction and representational compensation across different levels by constructing lightweight multi-scale enhancement modules [39]. PRCII-Net, for example, proposed a cross-scale information interaction feature pyramid, termed CII-FPN [7]. This module enhances the use of shallow and deep features while strengthening interaction among deep features, thereby improving small object detection in complex underwater environments. Similarly, Dynamic YOLO [8] improves the efficiency of multi-scale representation integration through a dynamic feature fusion network. These studies suggest that breaking away from fixed-path propagation and promoting stronger cross-scale interaction is an effective way to improve feature integration quality and detection performance.

However, most existing multi-scale fusion methods still rely on fixed topologies or weakly selective aggregation. In degraded underwater scenes, such designs often propagate useful target cues and irrelevant background responses together, which makes small object information easier to dilute during fusion. This suggests that the key limitation of current fusion strategies lies not only in cross-level interaction itself, but also in the lack of content-aware branch selection. A more suitable fusion mechanism should be able to choose scale responses according to the input and refine the fused representation afterward. This observation motivates the need for a fusion strategy that performs adaptive selection before recalibration.

## 3. Methods

To address the challenges of blurred object contours, weakened textures, and the tendency of small objects to be lost in underwater scenes, we introduce WEC into the backbone. WEC establishes a sequential enhancement path in which structure-sensitive information is first compensated in the frequency domain and then refined in the spatial domain. To mitigate the dilution of informative features caused by indiscriminate propagation of multi-scale responses in complex backgrounds, we further introduce SSF into the neck. SSF adaptively selects responses from different receptive fields and then recalibrates the fused feature along the channel and spatial dimensions, enabling more precise aggregation of target-related information while suppressing background noise. The remaining components of the network follow the original YOLOv11 configuration [14], thereby preserving the stability and efficiency of the baseline architecture.

**Figure 1:**
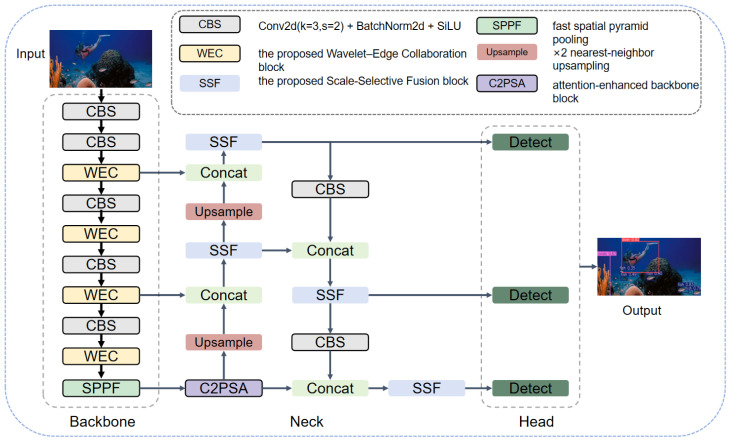
Overall architecture of WEC-UOD. WEC is embedded in the backbone for structure enhancement, and SSF is introduced in the neck for selective multi-scale fusion.

As shown in Figure 1, WEC-UOD follows a stage-specific design. In the backbone, WEC performs frequency-domain compensation before edge-guided spatial correction to recover structure-sensitive features. In the neck, SSF first selects informative receptive-field responses and then recalibrates the fused representation. Thus, the two modules address different stages of detector-internal representation learning rather than simply stacking existing operators.

### 3.1. Wavelet–Edge Collaboration

Under degraded underwater conditions, key structural cues such as object contours, boundaries, and weak textures are prone to progressive attenuation during deep feature extraction, while complex background clutter can easily induce false responses unrelated to the target [11]. Although directly embedding frequency-domain transformation or edge guidance into each basic unit may strengthen local structural responses, it also tends to entangle structural enhancement with basic representation learning, thereby increasing the complexity of the internal information flow and reducing optimization stability [1]. The proposed WEC module therefore introduces structural enhancement only after the main feature transformation and fusion stages. This design preserves the integrity of the original representation learning process while enabling a progressive refinement of structure-sensitive information through frequency-domain compensation followed by spatial correction.

**Figure 2:**
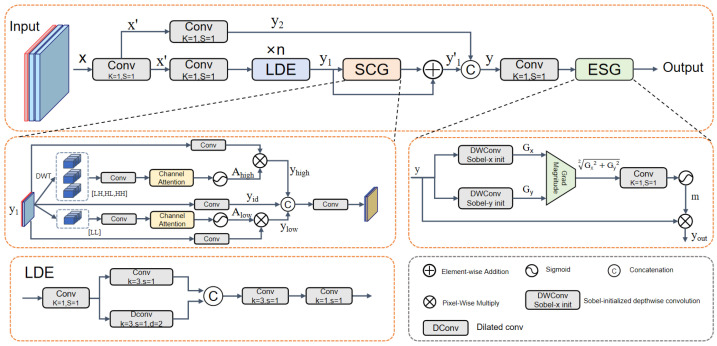
Structure of the proposed WEC module. It combines wavelet subband-guided compensation with edge-guided spatial refinement to enhance degraded structural features.

As shown in Figure 2, WEC follows a CSP-style design [40] with a shortcut branch and a transformation branch. The transformation branch is responsible for basic feature extraction and subsequent structural enhancement, whereas the shortcut branch preserves the original information flow. In this way, degraded textures and high-frequency details are first compensated in the frequency domain, and the resulting fused feature is then refined in the spatial domain to sharpen geometric boundaries.

The input feature is first projected by a 1×1 convolution and then split into two paths. In the transformation branch, *n* lightweight local detail extraction (LDE) units are stacked to learn the basic representation. Each LDE unit consists of a 1×1 convolution for channel reduction, two parallel 3×3 convolutions with dilation rates of 1 and 2 for local detail and limited context modeling, respectively, and a subsequent fusion path composed of 3×3 and 1×1 convolutions for feature integration and channel restoration. This design provides a reliable feature basis $y_{1}$ for subsequent structural enhancement at relatively low computational cost.

To recover degraded high-frequency details in the frequency domain, a Subband Cooperative Guidance (SCG) module is introduced at the end of the transformation branch. This module does not alter the spatial resolution of the feature map. Instead, it injects frequency-domain priors into the main branch in a residual manner, producing an intermediate feature representation with enhanced texture compensation. The compensated main branch feature is then fused with the shortcut branch feature and passed to an Edge-guided Spatial Correction (ESG) module for further refinement. In ESG, the gradient response is computed on the fused feature representation after SCG-based subband compensation, enabling the spatial gating process to further refine wavelet-enhanced structural cues. Using explicit gradient responses as spatial gating signals, ESG sharpens geometric boundaries, suppresses background noise, and produces the enhanced output feature $y_{\mathrm{out}}$.

Through this serial design, WEC refines the representation from basic feature extraction to frequency-domain compensation and then to spatial correction. Unlike a parallel use of frequency and edge cues, ESG operates on the feature representation already compensated by SCG, allowing edge-guided refinement to act on more reliable structural responses.

Let the input feature tensor be $x\in R^{B\times C_{0}\times H_{0}\times W_{0}}$, where *B*, $C_{0}$, $H_{0}$, and $W_{0}$ denote the batch size, channel number, feature-map height, and feature-map width, respectively. WEC first applies a 1×1 convolution, denoted by ${\mathrm{Conv}}_{1\times 1}\left(\cdot \right)$, to perform channel projection and obtain the transformed feature $x^{\prime }$. The resulting feature is then divided into a transformation branch and a shortcut branch. The transformation branch, denoted by T(·), extracts a basic representation $y_{1}$, while the shortcut branch, denoted by I(·), preserves the original information flow as $y_{2}$:

(1) $$x^{\prime }={\mathrm{Conv}}_{1\times 1}\left(x\right),\;y_{1}=T\left(x^{\prime }\right),\;y_{2}=I\left(x^{\prime }\right)$$

To account for the severe loss of high-frequency textures in underwater imaging, while low-frequency contour information remains relatively stable, we introduce the SCG module at the end of the transformation branch. Implemented in a residual manner, SCG injects frequency-domain priors into the main branch to compensate for the missing structural details in the basic representation $y_{1}$.

(2) $$y_{1}^{\prime }=y_{1}+G_{w}\left(y_{1}\right)$$

where $G_{w}\left(\cdot \right)$ denotes the proposed SCG module, which explicitly performs frequency-band decoupling of feature representations through the discrete wavelet transform (DWT).

Specifically, given the input feature $y_{1}$, a 2D DWT is first applied to decompose it into one low-frequency subband (LL), which preserves the main structural information, and three high-frequency subbands (LH, HL, and HH), which encode edge and texture details:

(3) $$\left(\;LL,LH,HL,HH\;\right)=DWT\left(y_{1}\right)$$

In this implementation, DWT is fixed as a single-level 2D Haar wavelet decomposition. The decomposition is applied independently to each channel of the feature map, producing one low-frequency subband and three directional high-frequency subbands. The Haar filters are non-learnable, and no multi-level DWT or alternative wavelet family is used in the reported experiments. Since single-level DWT reduces the spatial resolution of each subband by a factor of two, the generated frequency attention maps are resized to the spatial size of the input feature before feature modulation.

The decomposed subbands provide complementary frequency-domain descriptions of the input feature. The LL subband mainly retains coarse structural context and region-level appearance information, whereas the high-frequency subbands LH, HL, and HH capture directional edge and fine-texture responses that are more sensitive to scattering and blur. Accordingly, SCG performs separate channel recalibration for the low-frequency and high-frequency components. This frequency-specific recalibration helps preserve the distinction between coarse structural context and degradation-sensitive detail responses during feature reintegration. In the high-frequency branch, the three directional subbands are first aggregated by a 1×1 convolution and then passed through a channel attention module to generate the weight map $A_{\mathrm{high}}$, which is used to enhance texture details and boundary responses weakened by degradation. In parallel, the low-frequency branch applies a 1×1 convolution and a channel attention module to the LL subband to produce $A_{\mathrm{low}}$, which is used to preserve coarse structural context while reducing background interference:

(4) $$A_{\mathrm{high}}=\sigma \;\left(\mathrm{CA}\;\left({\mathrm{Conv}}_{1\times 1}\;\left([LH,HL,HH]\right)\right)\right),\;A_{\mathrm{low}}=\sigma \;\left(\mathrm{CA}\;\left({\mathrm{Conv}}_{1\times 1}\;\left(LL\right)\right)\right)$$

where CA(·) denotes the channel attention operation, σ(·) denotes the Sigmoid activation function, and all convolution kernels in this stage are of size 1×1.

To incorporate the generated frequency-domain priors into the spatial representation, the input feature $y_{1}$ is projected into three components, namely an identity component, a high-frequency-guided component, and a low-frequency-guided component, which are modulated by the corresponding weights, as described in Equation (5). The identity component preserves the original response, while the other two components introduce frequency-specific modulation for detail enhancement and structural context adjustment. The three components are then concatenated and fused by a 1×1 convolution, yielding the frequency-compensated feature $G_{w}\left(y_{1}\right)$ in Equation (6).

(5) $$y_{\mathrm{id}}=P_{\mathrm{id}}\left(y_{1}\right),\;y_{\mathrm{high}}=P_{\mathrm{high}}\left(y_{1}\right)⊙A_{\mathrm{high}},\;y_{\mathrm{low}}=P_{\mathrm{low}}\left(y_{1}\right)⊙A_{\mathrm{low}}$$

(6) $$G_{w}\left(y_{1}\right)={\mathrm{Conv}}_{1\times 1}\;\left([y_{\mathrm{id}},y_{\mathrm{high}},y_{\mathrm{low}}]\right)$$

Here, $y_{\mathrm{id}}$, $y_{\mathrm{high}}$, and $y_{\mathrm{low}}$ denote the identity component, the high-frequency-guided component, and the low-frequency-guided component, respectively. $P_{\mathrm{id}}\left(\cdot \right)$, $P_{\mathrm{high}}\left(\cdot \right)$, and $P_{\mathrm{low}}\left(\cdot \right)$ denote the 1×1 convolution-based channel-alignment mappings, and ⊙ denotes element-wise multiplication.

After the main branch is compensated, WEC fuses the compensated main branch feature $y_{1}^{\prime }$ with the shortcut branch feature $y_{2}$ to obtain the fused representation *y*, which is formulated as follows:

(7) $$y={Conv}_{1\times 1}\left(\left[y_{1}^{\prime },\;y_{2}\right]\right)$$

To further strengthen geometric boundaries and fine-grained structural information, WEC introduces ESG at the fusion output stage. Given the fused feature y, ESG uses explicit gradient responses as spatial gating signals to perform boundary-aware spatial refinement. Specifically, *y* is fed into two depthwise convolution branches initialized with horizontal and vertical Sobel kernels, respectively, to extract the directional gradient responses along the *x*- and *y*-directions [41]. The resulting responses are further combined to obtain the gradient magnitude:

(8) $$G_{x}={\mathrm{DWConv}}_{x}\left(y\right),\;G_{y}={\mathrm{DWConv}}_{y}\left(y\right),\;G=\sqrt{G_{x}^{2}+G_{y}^{2}}$$

where DWConv_*x*_(·) and DWConv_*y*_(·) denote 3×3 depthwise convolution operators initialized with horizontal and vertical Sobel kernels, respectively. They are implemented with stride 1, padding 1, and the number of groups equal to the number of input channels. The Sobel kernels are used only for initialization and are updated during training. *G* denotes the aggregated gradient magnitude response.

(9) $$m=\sigma \;\left({\mathrm{Conv}}_{1\times 1}\left(G\right)\right)$$

The edge response map *m* is then used to modulate the fused feature y in an element-wise manner, yielding the ESG-refined output:

(10) $$y_{out}=y⊙m$$

The frequency-domain and edge-guided components in WEC complement each other. Subband decomposition introduces structure-aware information from different frequency ranges, while ESG further sharpens geometric boundaries on the fused feature map through explicit gradient-based spatial refinement. As a result, the features delivered to the neck preserve clearer contours and boundary structures, which is beneficial for subsequent multi-scale aggregation.

### 3.2. Scale-Selective Fusion

**Figure 3:**
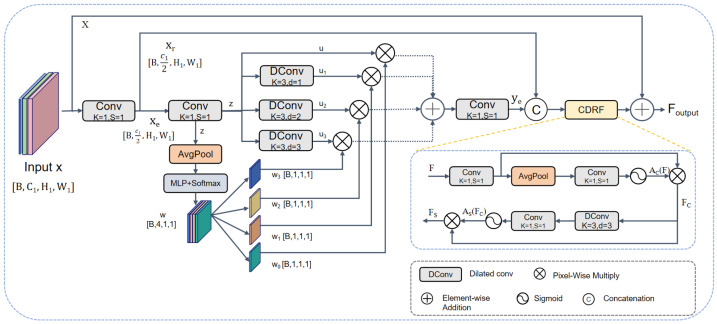
Structure of the proposed SSF module. It performs adaptive scale selection and fusion, followed by channel and spatial recalibration.

Although WEC improves single-level structural representation, multi-scale fusion is still needed to handle small objects and large shape variations in underwater scenes. However, features from different levels do not contribute equally: shallow features preserve more details, whereas deeper features provide stronger semantics and broader context. If they are fused in a fixed or uniform manner, useful target information and background noise are likely to be propagated together. To address this issue, SSF first selects responses from branches with different receptive fields according to the input feature and then refines the fused result through channel and spatial recalibration. As illustrated in Figure 3, the core of SSF lies in scale-selective aggregation, while a lightweight channel–spatial refinement is applied afterward to stabilize the fused representation.

In the partition-based modeling stage, let the input feature tensor be $x\in R^{B\times C_{1}\times H_{1}\times W_{1}}$, where *B*, $C_{1}$, $H_{1}$, and $W_{1}$ denote the batch size, channel number, feature map height, and feature map width, respectively. The input feature is first transformed by a convolutional mapping and then evenly split along the channel dimension into two parts, $x_{e}$ and $x_{r}$, where $x_{e},x_{r}\in R^{B\times \frac{C_{1}}{2}\times H_{1}\times W_{1}}$. The branch $x_{e}$ is sent to the pyramid aggregation path to extract multiple receptive fields, while $x_{r}$ is retained to preserve a fine-grained feature path.

The next stage performs scale-selective aggregation. SSF first applies a 1×1 convolution to $x_{e}$ to obtain an intermediate representation *z*. Based on *z*, SSF then constructs N=4 parallel branches, including one identity branch and three depthwise-separable dilated-convolution branches with different receptive fields:

(11) $$u_{0}=z,\;u_{i}={\mathrm{DConv}}_{3\times 3,\;d_{i}}\left(z\right),\;d_{i}\in \left\{1,2,3\right\},\;i=1,2,3$$

where $u_{0}$ preserves local detail information, while the other branches capture contextual responses over progressively enlarged spatial ranges.

To enable adaptive branch selection, a global descriptor is first extracted from *z* by global average pooling, denoted by GAP(·), and then mapped by a lightweight multilayer perceptron, denoted by MLP(·), to a four-dimensional branch-weight vector. After Softmax normalization, the branch weights are obtained as

(12) $$w=\mathrm{Softmax}\;\left(\mathrm{MLP}\;\left(\mathrm{GAP}(z)\right)\right),\;w=\left[w_{0},w_{1},w_{2},w_{3}\right]$$

where $w_{i}$ denotes the normalized weight assigned to the *i*-th branch. Accordingly, the aggregated feature is computed by weighted summation over the four parallel branches, followed by a 1×1 convolution:

(13) $$y_{e}={\mathrm{Conv}}_{1\times 1}\;\left(\sum_{i=0}^{3}w_{i}u_{i}\right)$$

To further refine the selected multi-scale representation, SSF first combines the scale-aggregated feature $y_{e}$ and the retained component $x_{r}$ through concatenation followed by a 1×1 convolution, yielding a joint representation *F*:

(14) $$F={\mathrm{Conv}}_{1\times 1}\;\left([y_{e},x_{r}]\right)$$

A channel–spatial dual recalibration fusion module (CDRF) is then applied to *F* to enhance informative responses and suppress irrelevant interference. Specifically, the channel attention map is generated from the global descriptor of *F* by global average pooling, followed by a 1×1 convolution and a Sigmoid activation:

(15) $$A_{c}\left(F\right)=\sigma \;\left({\mathrm{Conv}}_{1\times 1}\;\left(\mathrm{GAP}(F)\right)\right)$$

Accordingly, the channel-refined feature is obtained as

(16) $$F_{c}=F⊙A_{c}\left(F\right)$$

On this basis, spatial recalibration is further applied to $F_{c}$. A spatial response map is generated by a depthwise 3×3 convolution followed by a 1×1 convolution and a Sigmoid activation:

(17) $$A_{s}\left(F_{c}\right)=\sigma \left({\mathrm{Conv}}_{1\times 1}\left({\mathrm{DWConv}}_{3\times 3}\left(F_{c}\right)\right)\right).$$

The spatially recalibrated feature produced by CDRF is formulated as

(18) $$F_{s}=F_{c}⊙A_{s}\left(F_{c}\right)$$

For residual preservation, the original SSF input *x* is added back through an identity shortcut. The final output of SSF is therefore given by

(19) $$F_{\mathrm{out}}=F_{s}+x$$

where *x* denotes the input feature used for residual preservation.

Overall, SSF performs adaptive branch selection before channel–spatial recalibration and residual preservation. Compared with fixed concatenation or uniform aggregation, this selection-before-recalibration design allows the module to emphasize useful receptive-field responses while suppressing background interference during multi-scale fusion.

## 4. Experiments

To evaluate WEC-UOD, we conducted comparative, ablation, and qualitative experiments on the datasets. These experiments were conducted to assess overall detection performance, the contributions of WEC and SSF, and the model’s behavior in challenging scenes involving small, blurred, or cluttered targets.

### 4.1. Datasets and Experimental Details

In the experiments, two representative underwater datasets, RUOD [42] and DUO [43], were selected for evaluation. The models were trained and tested independently on each dataset to evaluate their robustness across datasets with different scene distributions. The RUOD dataset contains 9800 training images and 4200 test images, covering 10 categories. It features common challenges in real underwater imaging, such as haze, color distortion, illumination interference, and background clutter, and therefore provides a realistic benchmark for evaluation. The DUO dataset consists of 6671 training images and 1111 test images, involving 4 typical categories of underwater objects. It contains diverse scenes with densely distributed targets, obvious scale variations, and blur, noise, and color distortion, making it suitable for evaluating detection performance in complex underwater environments.

To ensure a fair and reproducible comparison, all reproduced models were trained and evaluated under the same hardware and software conditions. The experiments were conducted on Ubuntu 20.04 LTS, using PyTorch 2.7.1, CUDA 11.8 (NVIDIA Corporation, Santa Clara, CA, USA), and an NVIDIA GeForce RTX 4090 GPU (NVIDIA Corporation, Santa Clara, CA, USA).Unless otherwise specified, the input resolution was set to 640×640 with a batch size of 32. All models were trained for 1000 epochs. The initial learning rate was set to 0.01 and decayed to $1\times 10^{-4}$, corresponding to a final learning rate factor of 0.01. The momentum was set to 0.937, the weight decay to $5\times 10^{-4}$, and a 3-epoch warm-up strategy was adopted. The random seed was fixed to 0 for all experiments.

Except for the proposed architectural modifications, the same YOLOv11 training pipeline was used for the baseline, the ablation variants, and WEC-UOD. The same data augmentation strategy was applied to all models, including mosaic augmentation, random affine transformation, random scaling, horizontal flipping, and HSV color-space augmentation. No additional dataset-specific augmentation, image enhancement preprocessing, test-time augmentation, or extra post-processing strategy was introduced. All compared models and ablation variants followed the same training schedule, augmentation configuration, and evaluation protocol to ensure a consistent comparison.

### 4.2. Evaluation Metrics

To objectively evaluate model performance, this study reports Precision (P), Recall (R), mAP@0.5, mAP@0.5:0.95, AP75, and scale-specific AP. The mAP@0.5 metric evaluates detection accuracy at an IoU threshold of 0.5, while mAP@0.5:0.95 averages AP over IoU thresholds from 0.5 to 0.95 with a step size of 0.05. AP75 is reported to examine localization quality under a stricter IoU threshold. In addition, AP_*S*_, AP_*M*_, and AP_*L*_ are used to evaluate performance on small, medium, and large objects, respectively.

The relationship between predictions and ground-truth annotations is described using True Positive (TP), False Positive (FP), and False Negative (FN). In object detection, a prediction is counted as a TP when its bounding box satisfies the specified IoU threshold with a ground-truth box and the predicted category is correct. For mAP@0.5, the IoU threshold is set to 0.5; for AP75, it is set to 0.75; and for mAP@0.5:0.95, AP is averaged over IoU thresholds from 0.5 to 0.95 with a step size of 0.05. A prediction is counted as an FP if it fails to match any ground-truth box under the specified IoU threshold or if the predicted category is incorrect. A ground-truth object that is not matched by any predicted box is counted as an FN. The IoU is defined as the ratio of the intersection area between the predicted box and the ground-truth box to their union area:

(20) $$\mathrm{IoU}=\frac{\left|B_{p}\cap B_{\mathrm{gt}}\right|}{\left|B_{p}\cup B_{\mathrm{gt}}\right|}$$

where $B_{p}$ and $B_{gt}$ denote the predicted box and the ground-truth box, respectively. Based on the above statistical quantities, Precision and Recall are defined as

(21) $$P\;=\;\frac{TP}{TP\;+\;FP}\;,\;R\;=\;\frac{TP}{TP\;+\;FN}$$

By varying the confidence threshold, a P-R curve can be obtained. For a given category, the Average Precision (AP) is defined as the area under the corresponding P-R curve:

(22) $$AP\;=\;\int_{0}^{1}P(R)dR$$

Accordingly, the mean average precision (mAP) is computed as the average AP over all categories:

(23) $$mAP\;=\;\frac{1}{K}\sum_{k=1}^{K}AP_{k}$$

where *K* denotes the number of categories in the dataset; specifically, K=10 for RUOD and K=4 for DUO. In the main comparison tables, mAP@0.5 is used as the primary overall metric for consistency with existing underwater object detection studies, while mAP@0.5:0.95, AP75, and scale-specific AP are further reported in the ablation studies to provide a stricter and more detailed evaluation.

### 4.3. Comparative Experiments

The proposed method was compared with a range of mainstream detection approaches. The selected comparison models include general-purpose object detectors, representative underwater-oriented detectors, and recent frequency-domain-based underwater detection methods. YOLOv11s [14] is used as the baseline because WEC-UOD is developed on this architecture. YOLOv5s [44], YOLOv8n [45], YOLOv8m [45], and YOLOv11l [14] are included to provide comparisons under different model capacities. Representative underwater-oriented methods, including PRCII-Net [7], GEE-UOD [5], AGS-YOLO [46], Bi2F-YOLO [47], and PDSC-YOLOv8n [48], are also selected. In addition, recent frequency-domain-based methods, including WDS-YOLO [29], UDINO [33], and UHF-UOD [49], are included for comparison.

#### 4.3.1. Comparison and Analysis on the RUOD Dataset

Table 1 shows the comparison results on the RUOD dataset. WEC-UOD achieves the best Precision, Recall, and mAP@0.5 among all compared methods, reaching 87.2%, 81.4%, and 87.4%, respectively. Compared with the YOLOv11s baseline, WEC-UOD improves Precision by 1.5 percentage points, Recall by 1.9 percentage points, and mAP@0.5 by 1.5 percentage points. The higher Recall shows that the proposed method reduces missed detections in degraded underwater scenes.

Compared with lightweight underwater detectors, WEC-UOD also achieves better detection accuracy. PRCII-Net, AGS-YOLO, PDSC-YOLOv8n, GEE-UOD, and UHF-UOD obtain mAP@0.5 values between 86.1% and 86.7%. WEC-UOD further improves mAP@0.5 to 87.4%. This result indicates that the proposed structure-sensitive feature enhancement provides additional benefits beyond lightweight attention or cross-scale fusion designs.

The comparison with frequency-domain-based methods also shows the advantage of WEC-UOD. WDS-YOLO, UHF-UOD, and UDINO obtain mAP@0.5 values of 85.6%, 86.5%, and 86.4%, respectively. WEC-UOD outperforms these methods. This suggests that frequency-domain modeling alone is not sufficient for underwater detection. The combination of wavelet-subband compensation and edge-guided spatial correction provides more useful structural cues.

The last several rows of Table 1 focus on models with comparable or higher computational scale. YOLOv8m and YOLOv11l have 25.8 M and 25.4 M parameters, respectively, which are close to the 25.0 M parameters of WEC-UOD. Their FLOPs are 78.7 G and 86.6 G, both higher than the 65.1 G FLOPs of WEC-UOD. However, their mAP@0.5 values are 86.3% and 86.4%, lower than that of WEC-UOD. Bi2F-YOLO has a larger model size and higher computational cost, with 32.3 M parameters and 98.9 G FLOPs. WEC-UOD improves mAP@0.5 by 0.6 percentage points over Bi2F-YOLO while using fewer parameters and FLOPs. These results show that the performance gain is not only caused by comparison with a smaller baseline. WEC-UOD remains competitive under a similar or larger computational budget.

To further evaluate whether a separate underwater image enhancement stage can improve downstream detection, WEC-UOD was compared with several conventional enhancement-then-detection pipelines on the RUOD dataset.

Table 2 compares WEC-UOD with several enhancement-then-detection pipelines. FUnIE-GAN + YOLOv12s decreases the detection accuracy compared with YOLOv12s. AST + YOLOv12s and UMCTN + YOLOv12s improve mAP@0.5 to 86.0% and 86.2%, respectively, but the gains remain limited. WEC-UOD achieves the best Precision, Recall, and mAP@0.5 in this comparison. This result shows that input-level enhancement does not always improve detection performance. A separate enhancement stage may change the data distribution or introduce artifacts. WEC-UOD avoids this additional stage and enhances structure-sensitive features inside the detector.

To provide a clearer class-level comparison, Table 3 reports the per-class AP@0.5 results of the baseline, GEE-UOD, and WEC-UOD on RUOD.

As shown in Table 3, WEC-UOD improves AP for ‘holothurian’, ‘echinus’, ‘scallop’, ‘starfish’, ‘fish’, and ‘corals’ compared with the YOLOv11s baseline. The gains are especially clear for ‘fish’ and ‘corals’, whose AP values increase by 8.5 and 3.4 percentage points, respectively. These categories often contain weak contours, partial boundaries, and low-contrast textures. Such degraded but recoverable structural cues can benefit from wavelet-subband compensation and edge-guided spatial correction.

The improvement is not uniform across all categories. Although ‘echinus’ improves over the baseline, its AP is still lower than that of GEE-UOD, indicating that the proposed structural enhancement does not dominate every category. AP decreases are also observed for ‘diver’, ‘cuttlefish’, ‘turtle’, and ‘jellyfish’. For ‘diver’, ‘cuttlefish’, and ‘turtle’, the baseline AP is already higher than 95%, leaving limited room for further improvement. The ‘jellyfish’ category is more challenging because its translucent appearance and diffuse boundaries provide less stable edge evidence. Therefore, explicit structure enhancement is more effective when recoverable contours or textures remain, but it may provide weaker guidance for categories with ambiguous boundaries or already saturated baseline performance. Overall, the gains on structurally degraded categories lead to the best mAP@0.5 of 87.4%, although the benefit is category-dependent.

**Figure 4:**
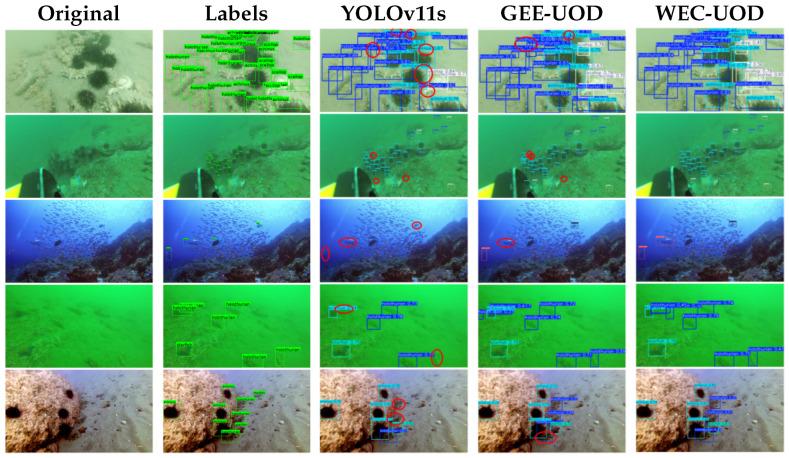
Qualitative comparison on representative RUOD scenes. Red circles highlight representative weak or small targets missed by YOLOv11s and GEE-UOD but successfully detected by WEC-UOD.

Figure 4 presents representative qualitative results on the RUOD dataset. WEC-UOD detects more weak and small targets than YOLOv11s and GEE-UOD in these examples. It also produces more complete responses in scenes affected by blur and background interference. The visual results are consistent with the quantitative results in Table 1 and Table 3.

#### 4.3.2. Comparison and Analysis on the DUO Dataset

Table 4 reports the comparison results on the DUO dataset. WEC-UOD achieves the best Recall and mAP@0.5, reaching 81.1% and 86.9%, respectively. Compared with the YOLOv11s baseline, WEC-UOD improves Precision by 1.0 percentage point, Recall by 0.9 percentage points, and mAP@0.5 by 1.2 percentage points. The improvement on DUO indicates that the proposed structure-enhanced representation remains effective in scenes with dense targets and large scale variation.

Compared with lightweight underwater detectors, WEC-UOD obtains higher detection accuracy. PRCII-Net, AGS-YOLO, PDSC-YOLOv8n, and GEE-UOD achieve mAP@0.5 values of 85.3%, 85.5%, 85.8%, and 86.1%, respectively. WEC-UOD improves the result to 86.9%. This shows that the proposed WEC and SSF modules can provide stronger feature representation for dense underwater object detection.

WEC-UOD also outperforms recent frequency-domain-based methods. WDS-YOLO, UHF-UOD, and UDINO obtain mAP@0.5 values of 85.6%, 86.1%, and 86.2%, respectively. These methods improve underwater detection by using frequency-related cues or degradation-aware modeling. In comparison, WEC-UOD achieves a higher mAP@0.5. This result indicates that wavelet-subband compensation can be more effective when it is further combined with edge-guided spatial correction and selective multi-scale fusion.

A similar trend can be observed under comparable or larger model complexity. Among the higher-capacity baselines, YOLOv8m and YOLOv11l obtain 84.4% and 86.2% mAP@0.5, respectively, while Bi2F-YOLO reaches 86.5% with 32.3 M parameters and 98.9 G FLOPs. WEC-UOD achieves a higher mAP@0.5 of 86.9% with 25.0 M parameters and 65.1 G FLOPs. This further supports that the improvement is not only caused by increasing model capacity.

For a class-wise comparison, Table 5 reports the AP@0.5 of each category on DUO for the baseline, GEE-UOD, and WEC-UOD.

As shown in Table 5, WEC-UOD achieves the best AP@0.5 in all four categories. Compared with the baseline, AP increases by 0.9, 0.7, 3.2, and 0.2 percentage points for ‘holothurian’, ‘echinus’, ‘scallop’, and ‘starfish’, respectively. The largest gain appears in ‘scallop’, which is usually more sensitive to low contrast, background clutter, and scale variation. The improvement suggests that WEC-UOD can preserve weak local structures and improve feature aggregation for small and dense objects.

For ‘starfish’ and ‘echinus’, the baseline AP is already high. The gains are therefore smaller, but WEC-UOD still achieves the best results. For ‘holothurian’, the improvement is moderate. This category often has elongated shapes and partial occlusion, where edge-guided correction can help refine local structure. Overall, the class-wise results show that WEC-UOD improves the DUO dataset in a stable manner, with the most obvious benefit appearing in the category with weaker appearance cues.

**Figure 5:**
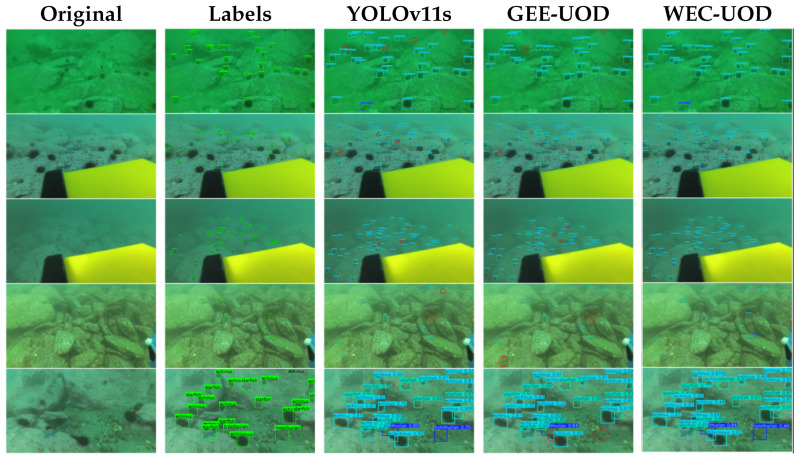
Qualitative comparison on the DUO dataset. Red circles indicate representative cases where WEC-UOD achieves more reliable detection than the compared methods.

Representative DUO samples are shown in Figure 5. As highlighted by the red circles, YOLOv11s and GEE-UOD miss several small or weak targets in cluttered and low-contrast scenes. WEC-UOD detects more of these targets and gives more complete responses in the selected examples. This visual comparison is consistent with the improvements in Recall and mAP@0.5 shown in Table 4.

Overall, the results on RUOD and DUO show that WEC-UOD improves underwater object detection across two datasets. The improvement is more pronounced for categories and scenes where weak but recoverable structural cues remain.

### 4.4. Ablation Experiments

To verify the effectiveness of the proposed components, ablation studies were conducted based on the YOLOv11s baseline under the same training strategy and experimental settings.

#### 4.4.1. Main Ablation Study on RUOD and DUO

This section analyzes the respective effects of WEC and SSF and examines whether the two modules provide complementary gains on the RUOD and DUO datasets. Although mAP@0.5 is reported as the main metric for comparison with existing underwater object detection methods, the analysis is not limited to this relatively loose IoU threshold. We further report mAP@0.5:0.95, AP75, and scale-specific AP to examine the contribution of each module under stricter localization criteria and across different object sizes.

On the RUOD dataset, the ablation results in Table 6 show that both WEC and SSF improve the YOLOv11s baseline when introduced individually. WEC increases mAP@0.5 from 85.9% to 87.1%, whereas SSF raises it to 86.5%. When the two modules are used together, WEC-UOD achieves the best overall result, reaching 87.4% mAP@0.5, 81.4% Recall, and 87.2% Precision. The improvement in Recall suggests that WEC-UOD helps reduce missed detections in degraded underwater scenes. This trend indicates that WEC is the main source of improvement on RUOD, while SSF provides an additional gain when combined with WEC.

To further examine whether the improvement remains under stricter evaluation criteria, Table 7 reports mAP@0.5:0.95, AP75, and scale-specific AP on RUOD. A similar pattern can be observed. Relative to the baseline, WEC improves mAP@0.5:0.95 from 61.7% to 62.9%, while SSF raises it to 62.3%. The full model further reaches 63.4%, which is the best result in this comparison. The same ranking is also maintained for AP75, where WEC-UOD improves the baseline from 68.2% to 69.6%. These results indicate that the gain is not only reflected under the relatively lenient mAP@0.5 metric, but also remains under stricter localization requirements.

The scale-specific results provide a more direct view of the behavior on objects of different sizes. WEC-UOD achieves the highest AP across small, medium, and large objects, reaching 18.2%, 48.4%, and 69.9%, respectively. Compared with YOLOv11s, the gains are 1.2 percentage points in AP_*S*_, 1.5 points in AP_*M*_, and 1.5 points in AP_*L*_. The improvement in AP_*S*_ suggests that the proposed modules can alleviate the loss of weak structural cues for small underwater objects. This is consistent with the design motivation of WEC and SSF: WEC enhances degradation-sensitive contours and texture responses, while SSF selectively aggregates multi-scale features to reduce the dilution of small-object information by background responses.

Table 8 reports the ablation results on the DUO dataset. As on RUOD, both WEC and SSF improve detection performance when introduced separately, with WEC producing the larger gain. Specifically, WEC increases mAP@0.5 from 85.7% to 86.5%, whereas SSF raises it to 86.3%. When the two modules are combined, WEC-UOD reaches the best overall result, with 86.9% mAP@0.5 and 81.1% Recall. The improvement in Recall is consistent with the RUOD results, suggesting that the proposed structure-enhanced representation helps reduce missed detections across different underwater datasets.

The extended results in Table 9 show that this tendency also holds under stricter metrics on DUO. Compared with the baseline, WEC raises mAP@0.5:0.95 from 61.2% to 62.0%, and SSF improves it to 61.8%. The full model reaches 62.5%, again giving the best result. The same ordering is observed for AP75, which increases from 67.8% for YOLOv11s to 69.1% for WEC-UOD. Therefore, the improvement is not restricted to mAP@0.5, but is also reflected in stricter localization metrics.

In terms of scale-specific AP, WEC-UOD achieves the best performance for small, medium, and large objects, with AP_*S*_, AP_*M*_, and AP_*L*_ of 19.9%, 48.0%, and 69.3%, respectively. Compared with the baseline, the gains are 1.2, 1.2, and 1.2 percentage points. These results indicate that WEC-UOD alleviates the loss of small-object cues under underwater degradation.

Overall, the ablation results on both datasets demonstrate a clear and consistent effectiveness of the proposed design. WEC contributes the main performance gain, showing that structure-sensitive compensation is essential for degraded underwater features. SSF further enhances the full model by selectively aggregating multi-scale responses, which helps preserve the structural cues recovered by WEC. The complete WEC-UOD consistently obtains the best results across mAP@0.5, mAP@0.5:0.95, AP75, and scale-specific AP. This indicates that the improvement is not limited to coarse detection accuracy, but also extends to stricter localization and objects of different scales. The results therefore provide direct evidence that WEC and SSF play complementary roles in improving underwater object detection. Although the absolute gains in mAP@0.5 are moderate, the improvements under stricter localization and scale-specific metrics further support the robustness of WEC-UOD under underwater degradation. In particular, WEC-UOD consistently improves AP75 and AP_*S*_ on both RUOD and DUO, indicating that the proposed modules enhance not only overall detection accuracy but also localization quality and small-object representation.

#### 4.4.2. Internal Ablation Study of WEC on RUOD

To further examine the respective roles and collaboration strategy of the two submodules within WEC, we conducted an internal ablation study on SCG and ESG on the RUOD dataset. The results are summarized in Table 10. Compared with the YOLOv11s baseline, both SCG and ESG improve detection performance when introduced separately. Specifically, SCG raises mAP@0.5 from 85.9% to 86.8%, while ESG improves it to 86.7%. This shows that frequency-domain compensation and edge-guided spatial correction are both useful for underwater feature enhancement.

To further distinguish the proposed ordered design from a simple integration of the two cues, we also evaluate a parallel concat variant. In this variant, SCG and ESG take the same input feature independently. Their outputs are concatenated along the channel dimension and then compressed by a 1×1 convolution to restore the original channel number. This variant obtains 86.8% mAP@0.5, which is better than ESG alone and comparable to SCG alone. However, it is still lower than the complete WEC module. The proposed WEC reaches the best result, with 87.1% mAP@0.5, 87.0% Precision, and 79.8% Recall.

These results indicate that directly combining subband compensation and edge-guided correction is not the most effective strategy. SCG provides the larger individual improvement, suggesting that subband-guided compensation plays the primary role in recovering degradation-sensitive structural information. ESG alone also brings a measurable gain, showing that explicit spatial correction remains helpful for refining local boundaries and suppressing background interference. When ESG is applied after SCG, the edge-guided correction operates on a compensated feature representation. This ordered compensation-correction process leads to better performance than the parallel concat variant. Therefore, SCG and ESG are complementary, and their sequential collaboration is more effective than simple parallel fusion.

To further inspect whether this tendency remains under stricter evaluation criteria and across different object sizes, Table 11 reports an extended comparison. A similar pattern can be observed. Relative to the baseline, SCG improves mAP@0.5:0.95 from 61.7% to 62.5%, while ESG raises it to 62.3%. The parallel concat variant further improves mAP@0.5:0.95 to 62.7%, indicating that a direct combination of the two cues can enhance detection quality under stricter IoU thresholds. However, the complete WEC module achieves the best result, reaching 62.9% mAP@0.5:0.95. The same trend is also observed for AP75, where the full WEC obtains 69.1%, higher than the parallel concat variant.

From the perspective of object scale, all variants improve AP_*S*_, AP_*M*_, and AP_*L*_ over the baseline. The parallel concat variant improves AP_*M*_ to 47.6%, but its AP_*S*_ and AP_*L*_ are lower than those of the full WEC. The complete WEC raises AP_*S*_ from 17.0% to 17.8%, AP_*M*_ from 46.9% to 47.9%, and AP_*L*_ from 68.4% to 69.3%. These results show that the proposed sequential design is more stable across object scales.

Overall, the internal ablation results indicate that SCG is the dominant contributor within WEC, while ESG provides additional spatial refinement. More importantly, the comparison with the parallel concat variant shows that the improvement does not simply come from using frequency-domain and edge cues at the same time. Although the parallel variant also uses both cues, its performance is still lower than that of the proposed sequential design. This suggests that the dependency between the two operations is important. Frequency-domain compensation first restores degradation-sensitive structural responses, and edge-guided correction then refines the compensated representation in the spatial domain. Therefore, WEC is better understood as an ordered compensation-correction mechanism rather than a simple aggregation of two enhancement operations.

#### 4.4.3. Internal Ablation Study of SSF on RUOD

To further analyze the internal design of SSF, we conduct an ablation study on the RUOD dataset. The results are summarized in Table 12. SSF contains two key operations: adaptive branch weighting and CDRF. The former assigns adaptive weights to different receptive-field branches, while the latter recalibrates the fused representation in the channel and spatial dimensions. To examine their respective effects, we evaluate two variants: SSF w/o adaptive weighting and SSF w/o CDRF.

In the SSF w/o adaptive weighting variant, the adaptive weights for different branches are removed. The multi-scale branch outputs are directly concatenated and compressed, while CDRF is retained for feature recalibration. This variant improves mAP@0.5 from 85.9% to 86.3%, showing that multi-scale receptive-field aggregation together with recalibration is useful for neck feature fusion. However, its performance is still lower than that of the full SSF, indicating that adaptive branch weighting provides an additional benefit by selecting more informative scale responses before fusion.

In the SSF w/o CDRF variant, adaptive branch weighting is retained, but the channel–spatial recalibration module is removed. This variant achieves 86.2% mAP@0.5, which is lower than SSF w/o adaptive weighting and the full SSF. This result indicates that CDRF plays an important role in refining the fused multi-scale representation and suppressing less useful responses after aggregation. The complete SSF obtains the best result, reaching 86.5% mAP@0.5, 86.4% Precision, and 78.9% Recall. These results show that adaptive branch weighting and CDRF are complementary, and that the full selection-before-recalibration design is more effective than either partial variant.

To further examine whether this trend remains under stricter evaluation criteria and across different object sizes, Table 13 reports mAP@0.5:0.95, AP75, and scale-specific AP on RUOD. SSF w/o adaptive weighting improves mAP@0.5:0.95 from 61.7% to 62.2% and AP75 from 68.2% to 68.4%. This indicates that direct multi-scale fusion with CDRF can already improve detection quality under stricter localization criteria. When CDRF is removed but adaptive branch weighting is retained, mAP@0.5:0.95 reaches 62.0% and AP75 reaches 68.3%. The lower result suggests that recalibration after scale aggregation is important for refining the fused representation.

The complete SSF achieves the best performance under all extended metrics, with 62.3% mAP@0.5:0.95 and 68.6% AP75. In terms of scale-specific AP, full SSF also obtains the highest AP_*S*_, AP_*M*_, and AP_*L*_, reaching 17.4%, 47.5%, and 68.8%, respectively. Compared with SSF w/o adaptive weighting, the full SSF further improves AP_*S*_ from 17.3% to 17.4%, AP_*M*_ from 47.4% to 47.5%, and AP_*L*_ from 68.7% to 68.8%. These results show that adaptive branch weighting brings additional gains when combined with CDRF, while CDRF remains important for stabilizing and refining the selected multi-scale features.

Overall, the internal ablation results show that the effect of SSF does not come only from adding multi-scale branches. The SSF w/o adaptive weighting variant still improves the baseline, but it is inferior to the full SSF, indicating that adaptive branch weighting helps select more useful receptive-field responses. The SSF w/o CDRF variant also improves the baseline, but its lower performance shows that channel–spatial recalibration is important after scale-aware aggregation. The full SSF achieves the best results because it first selects informative responses from different receptive-field branches and then recalibrates the fused representation. This supports the role of SSF as a selective multi-scale aggregation module rather than a simple feature concatenation operation.

## 5. Discussion

The experimental results on RUOD and DUO show that WEC-UOD improves underwater object detection, but the improvement is not evenly distributed across all categories and scenes. The ablation studies indicate that WEC contributes the main performance gain, while SSF further improves the full detector by selectively aggregating multi-scale features. This suggests that structure-sensitive feature recovery is important for detection under underwater degradation. Together with the internal WEC ablation, these results further show that the benefit of WEC-UOD is closely related to the ordered compensation-correction process. In this process, edge-guided refinement operates on frequency-compensated features rather than directly on the original degraded representation.

The proposed method is more beneficial when the target contains recoverable structural cues, such as weakened contours, partial boundaries, and low-contrast textures. This explains the clearer gains on categories such as ‘fish’ and ‘corals’ on RUOD, as well as ‘scallop’ on DUO. These categories are often affected by low contrast, background clutter, and scale variation, but their local shapes and texture responses can still be enhanced. In such cases, SCG compensates degradation-sensitive subband responses, ESG refines local spatial structures, and SSF helps preserve the recovered cues during multi-scale feature aggregation. The qualitative results also support this observation, where WEC-UOD detects more small or weak targets in cluttered and low-contrast scenes.

The advantage becomes less stable in two types of cases. First, for categories whose baseline AP is already high, such as ‘diver’, ‘cuttlefish’, and ‘turtle’, the remaining improvement space is limited. Therefore, the proposed structural enhancement may not lead to further AP gains for every high-confidence category. Second, for objects with intrinsically ambiguous boundaries, such as ‘jellyfish’, the available edge evidence can be weak or unstable because of translucent appearance and diffuse contours. In these cases, edge-guided spatial correction may provide limited benefit, and the enhanced structural responses may not always match the true object extent. This explains why WEC-UOD improves the overall mAP while still showing category-dependent variations.

These observations clarify the application boundary of WEC-UOD. The method is most effective for degraded but recoverable target structures, especially when weak contours and texture cues are still present. Its advantage may decrease when severe blur, turbidity, or backscatter removes most reliable structural evidence. Extremely small or heavily overlapped targets also remain challenging, because their discriminative cues can be suppressed before feature compensation and fusion. WEC-UOD requires more parameters and FLOPs than the YOLOv11s baseline. However, comparisons with YOLOv8m, YOLOv11l, and Bi2F-YOLO show that its improvement cannot be simply attributed to model scaling, as WEC-UOD remains competitive under comparable or larger computational budgets. Future work will focus on improving robustness in severely degraded underwater scenes and reducing model complexity while preserving structure-enhanced representation learning.

## 6. Conclusions

In this study, we presented WEC-UOD, a framework that enhances detector-internal representation learning rather than relying on an independent image enhancement preprocessing stage. To address the attenuation of structure-sensitive information in degraded underwater images and the limitations of non-selective multi-scale fusion, WEC was introduced into the backbone for wavelet–edge collaborative enhancement, and SSF was incorporated into the neck for selective multi-scale aggregation. Experimental results on the RUOD and DUO datasets show that WEC-UOD consistently outperforms the YOLOv11s baseline, achieving mAP@0.5 scores of 87.4% and 86.9%, respectively. Ablation studies further indicate that WEC contributes most of the performance gain, while SSF provides additional improvement by refining cross-scale feature fusion. Overall, the proposed design improves the detection of small objects and weak-texture targets in complex underwater scenes. Future work will focus on further improving the efficiency and robustness of the framework under more challenging underwater conditions.

## Figures and Tables

**Table 1:** Comparison results on the RUOD dataset. Bold and underlined values indicate the best results.

Model	Params (M)	FLOPs (G)	P (%)	R (%)	mAP@0.5 (%)
YOLOv11s [14] (baseline)	9.5	22.0	85.7	79.5	85.9
YOLOv5s [44]	9.1	24.0	83.7	78.2	83.9
YOLOv8n [45]	3.0	8.1	84.2	76.2	83.1
PRCII-Net [7]	2.9	7.8	86.3	78.9	86.5
AGS-YOLO [46]	3.0	9.6	86.1	79.6	86.4
PDSC-YOLOv8n [48]	5.6	8.9	86.2	78.6	86.1
GEE-UOD [5]	10.7	27.6	86.4	79.8	86.7
WDS-YOLO [29]	3.2	11.4	85.5	79.2	85.6
UHF-UOD [49]	11.7	24.9	86.4	78.6	86.5
UDINO [33]	69.5	41.4	86.8	79.1	86.4
YOLOv8m [45]	25.8	78.7	86.5	80.6	86.3
YOLOv11l [14]	25.4	86.6	86.6	79.5	86.4
Bi2F-YOLO [47]	32.3	98.9	86.8	80.6	86.8
WEC-UOD	25.0	65.1	**87.2 **	**81.4**	**87.4**

**Table 2:** Comparison with traditional enhancement-then-detection pipelines on the RUOD dataset. Bold and underlined values indicate the best results.

Model	Params (M)	FLOPs (G)	P (%)	R (%)	mAP@0.5 (%)
YOLOv11s [14] (baseline)	9.5	22.0	85.7	79.5	85.9
YOLOv12s [50]	9.28	21.69	85.6	79.3	85.8
FUnIE-GAN [51] + YOLOv12s [50]	16.3	31.9	84.7	77.5	84.7
AST [52] + YOLOv12s [50]	15.8	32.1	86.1	78.9	86.0
UMCTN [53] + YOLOv12s [50]	15.2	31.6	86.4	78.8	86.2
WEC-UOD	25.0	65.1	**87.2**	**81.4**	**87.4**

**Table 3:** Per-class AP@0.5 (%) results of the baseline, GEE-UOD, and the proposed method on the RUOD dataset. Bold and underlined values indicate the best results.

Class	Baseline (YOLOv11s) [14]	GEE-UOD [5]	Ours
Holothurian	80.2	82.2	**83.9 **
Echinus	90.8	**92.1**	91.6
Scallop	80.4	82.5	**84.9**
Starfish	90.1	90.4	**91.4**
Fish	69.1	69.6	**77.6**
Corals	73.8	75.5	**77.2**
Diver	95.7	**95.9**	94.1
Cuttlefish	**98.1**	97.5	96.7
Turtle	**97.6**	97.4	96.7
Jellyfish	83.0	**84.1**	79.9
mAP@0.5 (%)	85.9	86.7	**87.4**

**Table 4:** Comparison results on the DUO dataset. Bold and underlined values indicate the best results.

Model	Params (M)	FLOPs (G)	P (%)	R (%)	mAP@0.5 (%)
YOLOv11s [14] (baseline)	9.5	22.0	85.8	80.2	85.7
YOLOv5s [44]	9.1	24.0	84.3	78.4	83.7
YOLOv8n [45]	3.0	8.1	84.5	78.8	83.8
PRCII-Net [7]	2.92	7.8	86.0	79.3	85.3
AGS-YOLO [46]	3.0	9.6	86.1	79.6	85.5
PDSC-YOLOv8n [48]	5.6	8.9	86.3	80.1	85.8
GEE-UOD [5]	10.7	27.6	86.4	80.7	86.1
WDS-YOLO [29]	3.2	11.4	86.1	79.9	85.6
UHF-UOD [49]	11.7	24.9	86.6	80.3	86.1
UDINO [33]	69.5	41.4	86.8	80.6	86.2
YOLOv8m [45]	25.8	78.7	85.2	79.5	84.4
YOLOv11l [14]	25.4	86.6	**86.9 **	80.6	86.2
Bi2F-YOLO [47]	32.3	98.9	**86.9**	80.9	86.5
WEC-UOD	25.0	65.1	86.8	**81.1**	**86.9**

**Table 5:** Per-class AP@0.5 (%) results of the baseline, GEE-UOD, and the proposed method on the DUO dataset. Bold and underlined values indicate the best results.

Class	Baseline (YOLOv11s) [14]	GEE-UOD [5]	Ours
Holothurian	84.7	84.8	**85.6 **
Echinus	93.1	92.7	**93.8**
Scallop	69.8	72.8	**73.0**
Starfish	95.0	94.1	**95.2**
mAP@0.5 (%)	85.7	86.1	**86.9**

**Table 6:** Ablation results on the RUOD dataset. Bold and underlined values indicate the best results.

Model	Params (M)	FLOPs (G)	P (%)	R (%)	mAP@0.5 (%)
Baseline (YOLOv11s) [14]	9.5	22.0	85.7	79.5	85.9
+WEC	23.5	57.6	87.0	79.8	87.1
+SSF	10.5	23.7	86.4	78.9	86.5
WEC-UOD	25.0	65.1	**87.2 **	**81.4**	**87.4**

**Table 7:** Extended ablation results on the RUOD dataset. Bold and underlined values indicate the best results.

Model	mAP@0.5 (%)	mAP@0.5:0.95 (%)	AP75 (%)	AP_*S*_ (%)	AP_*M*_ (%)	AP_*L*_ (%)
YOLOv11s [14]	85.9	61.7	68.2	17.0	46.9	68.4
+WEC	87.1	62.9	69.1	17.8	47.9	69.3
+SSF	86.5	62.3	68.6	17.4	47.5	68.8
WEC-UOD	**87.4**	**63.4**	**69.6**	**18.2**	**48.4**	**69.9**

**Table 8:** Ablation results on the DUO dataset. Bold and underlined values indicate the best results.

Model	Params (M)	FLOPs (G)	P (%)	R (%)	mAP@0.5 (%)
Baseline (YOLOv11s) [14]	9.5	22.0	85.8	80.2	85.7
+WEC	23.5	57.6	86.6	81.0	86.5
+SSF	10.5	23.7	86.4	80.8	86.3
WEC-UOD	25.0	65.1	**86.8**	**81.1**	**86.9**

**Table 9:** Extended ablation results on the DUO dataset. Bold and underlined values indicate the best results.

Model	mAP@0.5 (%)	mAP@0.5:0.95 (%)	AP75 (%)	AP_*S*_ (%)	AP_*M*_ (%)	AP_*L*_ (%)
YOLOv11s [14]	85.7	61.2	67.8	18.7	46.8	68.1
+WEC	86.5	62.0	68.7	19.5	47.6	68.9
+SSF	86.3	61.8	68.4	19.2	47.3	68.6
WEC-UOD	**86.9**	**62.5**	**69.1**	**19.9**	**48.0**	**69.3**

**Table 10:** Internal ablation results of WEC on the RUOD dataset. Bold and underlined values indicate the best results.

Model	Params (M)	FLOPs (G)	P (%)	R (%)	mAP@0.5 (%)
Baseline (YOLOv11s) [14]	9.5	22.0	85.7	79.5	85.9
+SCG	22.4	56.2	86.6	79.7	86.8
+ESG	23.5	57.3	86.4	79.3	86.7
+SCG+ESG parallel concat	23.8	57.9	86.7	79.7	86.8
+WEC (SCG→ESG)	23.5	57.6	**87.0**	**79.8**	**87.1**

**Table 11:** Extended internal ablation results of WEC on RUOD. Bold and underlined values indicate the best results.

Model	mAP@0.5 (%)	mAP@0.5:0.95 (%)	AP75 (%)	AP_*S*_ (%)	AP_*M*_ (%)	AP_*L*_ (%)
YOLOv11s [14]	85.9	61.7	68.2	17.0	46.9	68.4
+SCG	86.8	62.5	68.8	17.6	47.6	69.0
+ESG	86.7	62.3	68.6	17.4	47.3	68.8
+SCG+ESG parallel concat	86.8	62.7	68.9	17.5	47.6	68.9
+WEC	**87.1**	**62.9**	**69.1**	**17.8**	**47.9**	**69.3**

**Table 12:** Internal ablation results of SSF on the RUOD dataset. Bold and underlined values indicate the best results.

Model	Params (M)	FLOPs (G)	P (%)	R (%)	mAP@0.5 (%)
Baseline (YOLOv11s) [14]	9.5	22.0	85.7	79.5	85.9
+SSF w/o adaptive weighting	10.3	23.5	86.3	78.8	86.3
+SSF w/o CDRF	10.3	23.4	86.1	78.7	86.2
+SSF	10.5	23.7	**86.4**	**78.9**	**86.5**

**Table 13:** Extended internal ablation results of SSF on RUOD. Bold and underlined values indicate the best results.

Model	mAP@0.5 (%)	mAP@0.5:0.95 (%)	AP75 (%)	AP_*S*_ (%)	AP_*M*_ (%)	AP_*L*_ (%)
YOLOv11s [14]	85.9	61.7	68.2	17.0	46.9	68.4
+SSF w/o adaptive weighting	86.3	62.2	68.4	17.3	47.4	68.7
+SSF w/o CDRF	86.2	62.0	68.3	17.2	47.1	68.5
+SSF	**86.5**	**62.3**	**68.6**	**17.4**	**47.5**	**68.8**

## Data Availability

The datasets used in this study are publicly available. The source code and reproduction scripts are available at https://github.com/liuofficial/WEC-UOD (accessed on 17 May 2026).
